# Successful control of an extended-spectrum beta-lactamase-producing *Klebsiella pneumoniae* ST307 outbreak in a neonatal intensive care unit

**DOI:** 10.1186/s12879-020-4889-z

**Published:** 2020-02-22

**Authors:** Eun-Hwa Baek, Se-Eun Kim, Sunjoo Kim, Seungjun Lee, Oh-Hyun Cho, Sun In Hong, Jeong Hwan Shin, Inyeong Hwang

**Affiliations:** 1 Department of Infection Control Team, Gyeongsang National University Changwon Hospital, Changwon, Republic of Korea; 2 Department of Laboratory Medicine, Gyeongsang National University Changwon Hospital, Changwon, Republic of Korea; 3 Department of Laboratory Medicine, Institute of Health Sciences, Gyeongsang National University College of Medicine, 15, Jinju-daero 816beon-gil, Jinju, 52727 Republic of Korea; 4 Department of Internal Medicine, Gyeongsang National University Changwon Hospital, Changwon, Republic of Korea; 5 Department of Laboratory Medicine, Inje University College of Medicine, Busan, Republic of Korea; 6 Busan Institute of Health and Environment, Busan, Republic of Korea

**Keywords:** Neonate, *Klebsiella pneumoniae*, Disease outbreak, Infection control, Molecular epidemiology, ST307

## Abstract

**Background:**

In this study, we evaluated the genetic relatedness of extended-spectrum beta-lactamase-producing *Klebsiella pneumoniae* (ESBL-KPN) isolates from an outbreak in a neonatal intensive care unit (NICU) in August 2017, We implemented an active countermeasure to control this outbreak successfully.

**Methods:**

The incidence of healthcare-associated ESBL-KPN bacteremia was evaluated before and after initiating enhanced infection control (IC) practices in January 2018. Surveillance cultures were set up and monitored for neonates, medical personnel, and NICU environments. Molecular analyses, including pulse-field gel electrophoresis (PFGE), sequence typing, and ESBL genotyping, were performed for the isolated KPN strains.

**Results:**

After implementing the enhanced IC procedures, the healthcare-associated bacteremia rate decreased from 6.0 to 0.0 per 1000 patient-days. Samples from neonates (*n* = 11/15, 73.3%), medical personnel (n = 1/41, 2.4%), and medical devices and the environments (6/181, 3.3%) tested positive for ESBL-KPN in the surveillance cultures in December 2017. Active surveillance cultures revealed that 23 of 72 neonates who were screened (31.9%) were colonized with ESBL-KPN between January and March 2018. All the isolates demonstrated closely related PFGE patterns and were identified as ST307 strain carrying the CTX-M-15 gene.

**Conclusions:**

Contaminated NICU environments and medical devices, as well as transmission by medical personnel, appeared to be the source of the outbreak of ESBL-KPN infection. We employed an enhanced IC strategy during January–March 2018 and successfully controlled the clonal outbreak of CTX-M-15-positive KPN. ST307 has emerged as an important bacteremia-causing pathogen in the NICU and should be carefully monitored.

## Background

The mortality and morbidity rates due to healthcare-associated infections (HAIs) are high in neonates who require intensive care because of their low immunity. In South Africa, the mortality from neonatal sepsis due to multi-drug resistant Enterobacteriaceae was reported to be 33.3% [1].

The neonatal intestines are a major reservoir of *Klebsiella pneumoniae* (KPN) [2], which is more transmissible than *Escherichia coli* [3]. As KPN is a normal component of the flora in stool specimens, it can easily contaminate a neonate’s surroundings and thereby cause a nosocomial outbreak. It is difficult to control such outbreaks caused by continuous shedding. Extended-spectrum beta-lactamase (ESBL) is an enzyme involved in the hydrolysis of third-generation cephalosporins as well as aztreonam. ESBL-producing KPN (ESBL-KPN) spreads by person-to-person contact or via environmental sources [4, 5]. Based on a systemic review of studies of ESBL-KPN outbreaks, the primary reservoirs of these pathogens include patients (48.9%), health-care workers (HCWs) (25.5%), and contaminated sinks (13.8%) [4].

KPN has the potential to survive in neonatal intensive care units (NICUs) for more than three years [5] and can re-emerge despite IC measures [3], and therefore, its eradication in the NICUs is challenging. Outbreaks of KPN, therefore, require enhanced infection control (IC) measures [6–8].

Infections with ESBL-KPN are associated with higher mortality and morbidity as well as increased lengths of stay and medical costs compared to those caused by KPN, which does not produce ESBL [9]. The prevalence of ESBL-KPN ranged from 22.8–52.9% in a children’s hospital in Korea [10, 11].

In this study, we evaluated the genetic relatedness of the ESBL-KPN isolates from this outbreak, using pulse-field gel electrophoresis (PFGE) and sequence typing, and found ST307 to be the causative strain in all cases. The ST307 strain has emerged as an important causal pathogen in the outbreaks of carbapenem-resistant Enterobacteriaceae [12, 13]. Most importantly, we successfully controlled the outbreak by the implementation of enhanced IC measures.

## Methods

### NICU setting

An ESBL-KPN bacteremia outbreak occurred in the 17-bed NICU of a university-affiliated hospital. This NICU, which is the referral center for the most severe cases in the region with one million population, has one isolation room with two beds and admits approximately 250 newborns each year. Staffed by three pediatricians and 35 nurses, it has a nurse-to patient ratio between 1:3 and 1:4. In 2017, the incidence of ESBL-KPN infections was 0.09/1000 patients-days for the whole institute and 0.17/1000 patients-days among the non-NICU children. This rate among the NICU neonates was 0.

Most of the neonates were premature infants who had respiratory distress, maternal risk of infections, or neonatal jaundice requiring intensive care. No routine screening for ESBL-KPN was performed before the outbreak because infections caused by this microorganism were absent in the NICU.

### Case definition

A case was defined as an infant either infected or colonized with ESBL-KPN. While infection was defined based on clinical and laboratory criteria according to the National Healthcare Safety Network definitions [14], colonization was defined as the presence of the causative agent in the absence of relevant symptoms. The incidence was calculated as the number of new cases per 1000 patient-days.

### Survey of the outbreak

Three bacteremia cases (two in neonates) involving ESBL-KPN were observed in August and September 2017. Considering the high risk of spread in the NICU, a reinforced conventional IC program was initiated in November 2017. However, three more cases were reported in December 2017. Since the incidence (6.0/1000 patients-days) exceeded the baseline rate, an outbreak was declared.

Accordingly, an extended infection control team (ICT) was organized to address this ESBL-KPN outbreak in the NICU. The ICT collaborated closely with the NICU team, and an outbreak survey was conducted immediately. First, the neonatal characteristics were determined. An individual record was filled out for each neonate which included demographic data, along with possible predisposing factors: gender, birth weight, age, duration of hospitalization, gestational age, Apgar score at five minutes, incubator care, use of antibiotics, mechanical ventilation, use of a central or peripheral venous catheter, location in the NICU, and presence of HAIs. Second, surveillance cultures were set up using samples from the neonates, HCWs, and the surroundings using a swab moisturized with sterile saline to identify potential reservoirs of ESBL-KPN. Active surveillance cultures (ASCs) were also set up to test the navel, axillary, inguinal, and perianal regions of the neonates (*N* = 17). Rectal swab specimens were also obtained from the HCWs in the NICU (*N* = 41) to screen for carriers. Samples from the surroundings (*N* = 110) and medical devices (*N* = 71) including incubators, total parenteral nutrition infusion pumps, glucometers, portable echocardiograms, patient monitors, respiratory therapy equipment, thermometers, stethoscopes, sinks, milk countertops, nursing carts, computer keyboards, telephones, doorknobs, and diaper scales were also cultured. To exclude the possibility of infections coming from the delivery room, samples from operation tables, incubators for emergency operations, childbirth boards, and birth measuring instruments were also cultured. Rectal swabs or stool specimens from 15 pregnant mothers who were admitted to the delivery room were cultured to rule out the vertical transmission of ESBL-KPN between January to February 2018. Third, infection control nurses monitored the compliance with hand hygiene practices among the medical personnel, and the contact precaution rates among HCWs.

### Reinforced and enhanced IC program

Improvements in hand hygiene and contact precautions were reinforced for all HCWs in the NICU. We also implemented a more frequent and thorough disinfection and cleaning of medical devices, incubators, and surroundings. Group education and frequent rounds of the NICU and other areas were performed to encourage IC activities and to emphasize the seriousness of the situation.

From January to March 2018, in addition to the reinforced IC program, an enhanced IC program was established, which included cohort care of neonates and medical personnel, active surveillance cultures (ASCs), and the requirement to wear gown and glove for medical services. The ASCs involved isolation of ESBL-KPN from the skin, fecal, or perianal specimens of neonates who did not have clinical symptoms or signs of infection. The ASCs were performed every week for neonates in the NICU until March 2018.

### Antibiotic susceptibility test and molecular epidemiological study

Bacteria were identified using MALDI-TOF MS (bioMérieux; Marcy l’Étoile, France), and antibiotic susceptibility tests were performed by the broth microdilution method using the Vitek-2 system (bioMérieux). Isolates of KPN showing ESBL resistance were analyzed by PFGE and multi-locus sequence typing to determine their genetic relatedness. Once the isolates were digested with *Xba*I (Roche, Basel, Switzerland) enzyme, electrophoresis was performed using CHEF MAPPER (Bio-Rad, Hercules, CA, USA). The agarose gel was stained with SYBR Gold (ThermoFisher Scientific, Waltham, MA, USA) to visualize the PFGE pattern. A dendrogram was obtained using BioNumerics (Bio-Rad) to evaluate the relationship between the strains. The similarity in PFGE patterns was interpreted according to the criteria of Tenover et al. [15]. Seven housekeeping genes (*rpoB*, *gapA*, *mdh*, *pgi*, *phoE*, *infB*, and *tonB*) were amplified and sequenced to identify STs as described at http://bigsdb.pasteur.fr/klebsiella/ [16]. The ESBL gene was amplified by PCR with known primers targeting the CTX-M-1, CTX-M-2, and CTX-M-9 genes [17, 18]. DNA sequencing was performed using the amplified PCR product, and ESBL genotypes were identified using BLAST.

### Statistical analysis

Differences in the incidence rates of ESBL-KPN infections between two months were tested using a two-sample z-test, which divided the logarithm of the ratio of the incidence rates by their estimated standard error using SPSS Statistics for Windows, version 24.0 (IBM Corp., Armonk, NY, USA). A *P* value < 0.05 was considered to be a significant difference. Differences in compliance with guidelines for hand hygiene and contact, before and after implementation of the enhanced IC measures were tested using the Fisher’s exact test.

## Results

### Neonates positive for ESBL-KPN

The clinical characteristics of the neonates who tested positive for ESBL-KPN are summarized in Table 1. All six neonates were premature with gestational ages and body weights ranging from 24 to 31 weeks, and 640–1505 g, respectively. One of them had reinfection. Importantly, while all the neonates had central venous catheters, four of them were on mechanical ventilation, and four had central line-associated bloodstream infections. Two neonates were exposed to ESBL-KPN positive HCWs. Despite the aggressive treatment, three (60%) of the five neonates with bacteremia died.

**Table 1:** Main characteristics of premature neonates colonized or infected with *Klebsiella pneumoniae* during the outbreak in 2017

Case	Sex	Interval^*^	Gestational age, (week + day)	Birth weight, (g)	Major invasive procedure	Type of infection	Positive culture specimens (date)	Exposure to RN 33^d^ (date)	Empirical antibiotic regimen	Outcome (cause of death)
1^b^	M	5.4	24 + 0	640	CVC, MV	CLABSI	B (8/15)	NO	VA, TZP, MER	Cured
2	F	39.4	31 + 5	1505	CVC, MV	CLABSI	U (9/1), B (9/8)	NO	VA, MER, CC	Death (sepsis)
3^c^	M	48.4	24 + 0	640	CVC, MV, Explorative laparotomy	CLABSI	B (9/27)	NO	VA, TZP, MER	Death (sepsis)
4	M	5.0	29 + 5	1150	CVC, MV, Explorative laparotomy	Colonized	S (11/1)	Yes (11/17–12/18)	VA, TZP, MER	Cured
5	F	13.5	26 + 6	1042	CVC	NEC, BSI	B (12/6)	Yes (12/21)	VA, TZP, MER	Cured
6	F	41.4	26 + 1	790	CVC, MV, PDA ligation	CLABSI	B (12/10)	NO	VA, TZP, MER	Cured
7	F	21.9	27 + 2	1215	CVC	CLABSI	B (12/29)	NO	VA, TZP, MER	Death (sepsis)

^**a**^ Interval from birth till when detected positive for ESBL-KPN

^**b**^ Index case

^**c**^ Cases 1 and 3 are of the same neonate

^**d**^ RN (registered nurse) 33 was positive in the rectal swab culture

*CVC* central venous catheter, *MV* mechanical ventilation, *PDA* patent ductus arteriosus, *CLABSI* central line-associated bloodstream infection, *NEC* necrotizing enterocolitis, *B* blood, *U* urine, *S* sputum, *VA* vancomycin, *TZP* piperacillin/tazobactam, *MER* meropenem, *CC* clindamycin

### Incidence

**Fig. 1:**
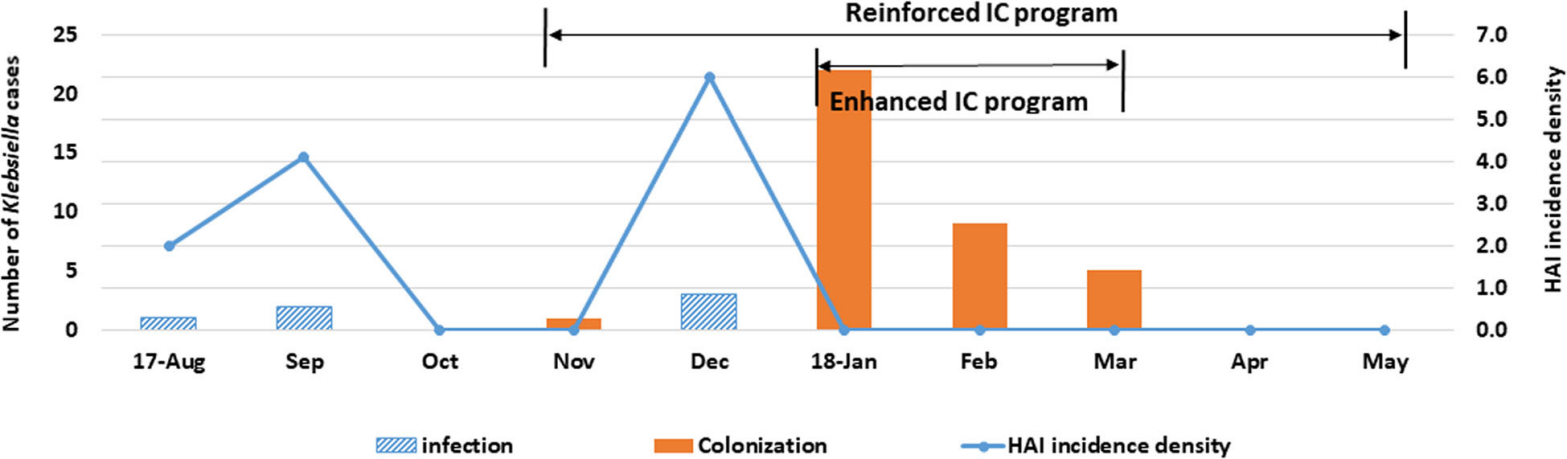
The incidence density of HAIs and distribution of the cases. Shown is the incidence of extended-spectrum beta-lactamase (ESBL)-producing *Klebsiella pneumoniae* (KPN) bacteremia in the neonatal intensive care unit. The reinforced IC practices included optimal hand hygiene, contact precautions, disinfection of medical devices, and cleaning the surroundings. The enhanced IC practices included cohort care of medical personnel and neonates, active surveillance cultures, and the use of disposable gowns and gloves for medical services in addition to the reinforced IC practices. HAI: healthcare-associated infections; IC: infection control

The incidence of bacteremia caused by ESBL-KPN in HAIs decreased from 6.0 to 0.0 after we implemented the enhanced IC measures (*P* = 0.026) (Fig. 1 & Supplementary File 1). While five cases of ESBL-KPN bacteremia were seen before the IC measures were implemented, there were none after the implementation of the measures. Of the five infected neonates, one had a re-infection. The number of neonates who were colonized with ESBL-KPN increased markedly from one before the implementation of IC measures to 36 after the enhanced measures were implemented. This increase was probably the result of better detection because of the routine screening tests performed during the enhanced IC period.

The incidence of ESBL-KPN infections detected by the ASCs decreased gradually from 45.0 to 25.5 and 18.5 in January, February, and March 2018, respectively. However, the differences in the incidence rates between two months (Jan vs. Feb and Feb vs. Mar) were not statistically significant (*P* = 0.146 and 0.560, respectively).

### Compliance with practices for infection prevention

Compliance with optimal hand hygiene practices increased from 60.8% (96/158) to 75.4% (303/402) (*P* = 0.001), and that with contact precaution measures also improved from 87.5% (42/48) to 98.3% (225/229) (*P* = 0.002) after initiation of the enhanced IC measures. The fractions indicate the positive performance over the observed indications for hand hygiene and contact precaution.

### Survey of the outbreak

Cultures of environmental samples and those collected from medical equipment in the delivery room were all negative (*N* = 28) for ESBL-KPN. Moreover, the rectal swabs and stool specimens from pregnant mothers (*N* = 15) tested also negative. Although one HCW (2.4%) tested positive in the surveillance culture, ESBL-KPN positivity was seen even after she resigned on January 8, 2018.

The ASCs found 11 neonates (73.3%) and six medical devices or incubators (3.3%) positive for ESBL-KPN. The mean duration of colonization by ESBL-KPN was 7.5 days (range: 3–50 days). These findings suggested that the neonates contracted ESBL-KPN from the medical devices or from the environment via the medical personnel.

No positive clinical specimens or HAIs were detected since January 2018, even after discontinuation of the enhanced IC measures until March 2018. We declared the end of the outbreak in June 2018. The number of neonates per nurse decreased from 1:3–1:4 to 1:2–1:3, thereby reducing the crowded conditions.

### Antibiotic resistance and molecular epidemiological characteristics

**Fig. 2:**
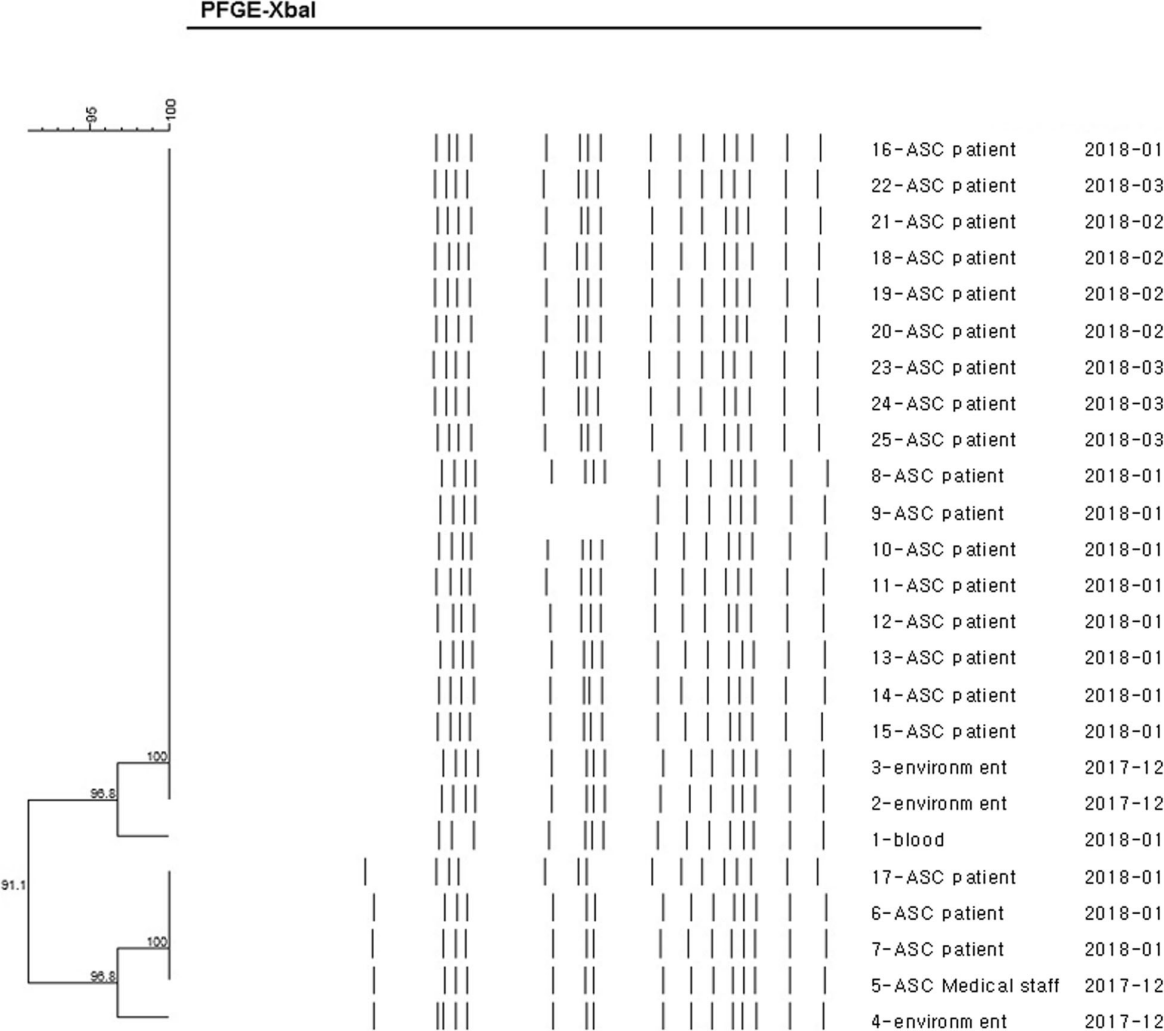
A dendrogram of the PFGE results for the *Klebsiella pneumoniae* isolates. All strains except those isolated from the blood, environment, and medical staff were obtained from the ASCs of the neonate skin surface, stool, or perianal swab specimens. All isolates show similar PFGE patterns. The 1-blood sample is from case 7 in Table 1, 2-environment sample is from the incubator of 8-ASC, 3-environment sample is from the surface of the washing room, and 4-environment sample is from 17-ASC. PFGE: pulsed-field gel electrophoresis; ASC: Active surveillance culture

All isolates were equally resistant to ampicillin, aztreonam, cefazolin, cefepime, ceftazidime, ciprofloxacin, gentamicin, and trimethoprim/sulfamethoxazole, but were susceptible to amikacin, cefoxitin, ertapenem, imipenem, piperacillin/tazobactam, and tigecycline. PFGE revealed that all strains isolated from the blood (*N* = 1), medical devices (*N* = 5), and neonate surveillance cultures (*N* = 13) were closely related (Fig. 2). The molecular type  identified in all the isolates was CTX-M-15 ESBL with ST307.

## Discussion

Preterm newborns are immunologically immature and often require invasive procedures, rendering them highly susceptible to infections [19, 20]. The risk of infections is associated with low birth weight, prolonged stay at the hospital, empirical antibiotic treatment, frequent manipulations, and nasopharyngeal or rectal colonization [2, 4, 5, 18, 20, 21]. A study from Spain found that the age of < 12 weeks and previous treatments with third-generation cephalosporins and aminoglycosides were associated with multi-drug resistant KPN colonization and infection [20]. The screening of high-risk patients during outbreaks is recommended to control epidemics [20]. As our study did not include a control group, we could not analyze the risk factors associated with the outbreak. We reduced the number of neonates admitted to the NICU during the outbreak to avoid conditions of over-crowding and under-staffing [7, 8].

In the first report of an ESBL-KPN outbreak in a NICU in Korea in 1996, isolates from the patients with sepsis showed the same PFGE patterns as seen in the ASC isolates from neonates and HCWs, indicating a clonal outbreak [22]. By implementing cohort care and strict barrier precautions, the outbreak was controlled. However, environmental cultures were not performed until a second outbreak occurred in the NICU in 2000, at which point ESBL-KPN had not yet been detected [6]. The origin of the outbreak was suspected to be an HCW who transmitted the pathogen to the neonates. The outbreak subsided when the neonates returned to the community [6]. Although in our study, a nurse tested positive at our institute, she was unlikely to have been the source of spread because the outbreak continued even after she resigned.

In a large-scale outbreak of ESBL-KPN, samples from 145 patients who were either infected or colonized with the agent showed the same clonal type, as determined by PFGE [2]. The carriage of ESBL-KPN in the digestive tract has been identified as the most important risk factor for ESBL-KPN infection or colonization [2]. Therefore, weekly rectal swabs should be tested to identify high-risk carriers in the NICU [2, 6]. There is also a significant correlation between the restricted use of oxyimino-*β-*lactams and the trends in ESBL-KPN infection. According to a previous study, a change to piperacillin/tazobactam from extended-spectrum cephalosporins decreased the prevalence of ESBL-KPN infections [11]. Therefore, the use of extended-spectrum cephalosporins should be restricted to control outbreaks of ESBL-KPN infection [20]. However, we did not apply an antibiotic stewardship program to control the outbreak.

Another large-scale outbreak of ST37 KPN occurred in Haiti in 2014–2015, accounting for 257 cases of sepsis and 191 deaths [23]. After improving the clinical management and strengthening infection prevention and control measures, the mortality rate dropped from 100 to 24%.

CTX-M-15 is prevalent in Korean hospitals, even in the bacteremia [24]. Several studies have reported the STs of ESBL-KPN from different outbreaks. For example, outbreaks have been caused by ST20 in Greece [25]; ST199 in Latvia [26]; ST14 in Tanzania [27]; ST13, ST16, ST35, ST48, and ST101 in France [28]; and ST607 in Spain [29]. In each of these studies, the CTX-M-15 ESBL type was reported.

ST307 has not been reported in NICU outbreaks to date. ST307 emerged in the mid-1990s, and its distribution has rapidly expanded worldwide. This strain is intimately associated with CTX-M-15 ESBL and sometimes exhibits carbapenem resistance [12, 30]. Therefore, close observation of the spread of ST307 is warranted. There was a clonal spread of ST307 KPN in Busan, Korea, in 2015, and the strain carried a self-transferable IncX3-type plasmid harboring bla_KPC-2_ [13]. In-depth genetic investigations, such as whole-genome sequencing, are needed to reveal the relationship between the clonal outbreak of 2015 and the one described in this study.

The reinforcement of hand hygiene is the most effective intervention to control outbreaks [4]. Therefore, we monitored the optimal hand hygiene rate as well as the contact precaution rate and found a significant increase in compliance after the implementation of the IC measures.

This study has several limitations. As the systematic microbiological screening was not continued or repeated after reinforcing the IC measures, we cannot be sure if the outbreak had really ended. There might have been undetected cases because the direct plating of rectal swabs has a low sensitivity of detection [31]. In addition, we did not have any cases of non-ESBL KPN during the same period, and therefore we could not perform a risk analysis. Moreover, we do not know clearly where this outbreak originated from. Colonization of this microorganism was very fast because the ASC of even a day old neonate tested positive.

We applied the IC measures in two steps. At the onset of the outbreak, reinforced IC measures were initiated. Optimal hand hygiene, contact precautions, disinfection of medical devices, and cleaning of the surroundings was monitored. Mutual collaboration between the ICT and medical personnel in the NICU or delivery room was promoted. The ICT provided the needed education and conducted frequent rounds of the NICU. Following the detection of three additional bacteremia cases, enhanced IC practices, including cohort care of neonates and attending nurses, use of disposable gloves and gowns for medical services, and ASCs were implemented, in addition to the reinforced IC measures. Finally, the outbreak was controlled 3 months after the implementation of the enhanced IC practices.

Molecular epidemiological studies were useful in characterizing the clonal outbreak and its mode of transmission. The strain of ESBL-KPN involved in this outbreak was CTX-M-15 with ST307. Our results highlight the potential of ST307 as a causative agent of bacteremia outbreaks in NICUs, as well as the importance of careful monitoring and control during such outbreaks.

## Conclusions

We describe an outbreak of ESBL-KPN bacteremia in a NICU and its successful control. Molecular epidemiological analyses demonstrated CTX-M-15 with ST307 to be the causative agent. We implemented enhanced IC practices and successfully controlled this outbreak.

## Supplementary information

**Additional file 1.** Case report form and raw data of Klebsiella pneumoniae outbreak, hand hygiene monitoring, and active surveillance cultures.

## Data Availability

The datasets used and/or analyzed during the current study are included in this published article and its supplementary information file.

## Abbreviations

*ASC*: Active surveillance cultures
*ESBL*: Extended-spectrum beta-lactamase
*HAI*: Healthcare-associated infection
HCW: Health-care workers
*IC*: Infection control
*ICT*: Infection control team
*KPN*: *Klebsiella pneumoniae*
*NICU*: Neonatal intensive care unit
*PFGE*: Pulse-field gel electrophoresis

## References

[1] Ballot DE, Bandini R, Nana T, Bosman N, Thomas T, Davies VA (2019). A review of -multidrug-resistant enterobacteriaceae in a neonatal unit in johannesburg, South Africa. BMC Pediatr.

[2] Pena C, Pujol M, Ardanuy C, Ricart A, Pallares R, Linares J (1998). Epidemiology and successful control of a large outbreak due to klebsiella pneumoniae producing extended-spectrum beta-lactamases. Antimicrob Agents Chemother.

[3] Artelt T, Kaase M, Bley I, Eiffert H, Mellmann A, Kuster H (2018). Transmission risk on a neonatal intensive care unit: Escherichia coli versus klebsiella pneumoniae. Can J Infect Dis Med Microbiol.

[4] Hendrik TC, Voor In ‘t Holt AF, Vos MC (2015). Clinical and molecular epidemiology of extended-spectrum beta-lactamase-producing klebsiella spp.: a systematic review and meta-analyses. PLoS One.

[5] Haller S, Eller C, Hermes J, Kaase M, Steglich M, Radonic A (2015). What caused the outbreak of esbl-producing klebsiella pneumoniae in a neonatal intensive care unit, Germany 2009 to 2012? Reconstructing transmission with epidemiological analysis and whole-genome sequencing. BMJ Open.

[6] Jun N-L, Kim M-N, Jeong J-S, Kim Y-S, Kim EA-R, Kim K-S (2006). Molecular-epidemiologic study on outbreak of colonization by extended spectrum β-lactamase producing klebsiella pneumoniae in neonatal intensive care unit. Korean J Pediatr.

[7] Kim MJ, Chung KS, Sohn KM (2013). Successful control of extended-spectrum beta-lactamase-producing klebsiella pneumoniae outbreak in a neonatal intensive care unit. Korean J Nosocomial Infect Control.

[8] Cantey JB, Sreeramoju P, Jaleel M, Trevino S, Gander R, Hynan LS (2013). Prompt control of an outbreak caused by extended-spectrum beta-lactamase-producing *Klebsiella pneumoniae* in a neonatal intensive care unit. J Pediatr.

[9] Stone PW, Gupta A, Loughrey M, Della-Latta P, Cimiotti J, Larson E (2003). Attributable costs and length of stay of an extended-spectrum beta-lactamase-producing klebsiella pneumoniae outbreak in a neonatal intensive care unit. Infect Control Hosp Epidemiol.

[10] Kim YK, Pai H, Lee HJ, Park SE, Choi EH, Kim J (2002). Bloodstream infections by extended-spectrum beta-lactamase-producing escherichia coli and klebsiella pneumoniae in children: epidemiology and clinical outcome. Antimicrob Agents Chemother.

[11] Lee J, Pai H, Kim YK, Kim NH, Eun BW, Kang HJ (2007). Control of extended-spectrum beta-lactamase-producing escherichia coli and klebsiella pneumoniae in a children's hospital by changing antimicrobial agent usage policy. J Antimicrob Chemother.

[12] Cejas D, Elena A, Guevara Nunez D, Sevillano Platero P, De Paulis A, Magarinos F (2019). Changing epidemiology of kpc-producing klebsiella pneumoniae in Argentina: emergence of hypermucoviscous st25 and high-risk clone st307. J Glob Antimicrob Resist.

[13] Kim JO, Song SA, Yoon EJ, Shin JH, Lee H, Jeong SH (2017). Outbreak of kpc-2-producing enterobacteriaceae caused by clonal dissemination of klebsiella pneumoniae st307 carrying an incx3-type plasmid harboring a truncated tn4401a. Diagn Microbiol Infect Dis.

[14] Centers for Disease Control and Prevention. https://www.cdc.gov/nhsn/pdfs/validation/2017/pcsmanual_2017.pdf.

[15] Tenover FC, Arbeit RD, Goering RV, Mickelsen PA, Murray BE, Persing DH (1995). Interpreting chromosomal DNA restriction patterns produced by pulsed-field gel electrophoresis: criteria for bacterial strain typing. J Clin Microbiol.

[16] Diancourt L, Passet V, Verhoef J, Grimont PA, Brisse S (2005). Multilocus sequence typing of klebsiella pneumoniae nosocomial isolates. J Clin Microbiol.

[17] Kim J, Lim YM, Jeong YS, Seol SY (2005). Occurrence of ctx-m-3, ctx-m-15, ctx-m-14, and ctx-m-9 extended-spectrum beta-lactamases in enterobacteriaceae clinical isolates in Korea. Antimicrob Agents Chemother.

[18] Song W, Kim J, Bae IK, Jeong SH, Seo YH, Shin JH (2011). Chromosome-encoded ampc and ctx-m extended-spectrum beta-lactamases in clinical isolates of proteus mirabilis from Korea. Antimicrob Agents Chemother.

[19] Villari P, Iacuzio L, Torre I, Scarcella A (1998). Molecular epidemiology as an effective tool in the surveillance of infections in the neonatal intensive care unit. J Inf Secur.

[20] Asensio A, Oliver A, Gonzalez-Diego P, Baquero F, Perez-Diaz JC, Ros P (2000). Outbreak of a multiresistant klebsiella pneumoniae strain in an intensive care unit: antibiotic use as risk factor for colonization and infection. Clin Infect Dis.

[21] Pessoa-Silva CL, Meurer Moreira B, Camara Almeida V, Flannery B, Almeida Lins MC, Mello Sampaio JL (2003). Extended-spectrum beta-lactamase-producing klebsiella pneumoniae in a neonatal intensive care unit: risk factors for infection and colonization. J Hosp Infect.

[22] Lee SH, Jeong J-S, Lee SY, Pai HJ, Nah J, Park SJ (1997). Outbreak of nosocomial infection caused by klebsiella pneumoniae producing extended-spectrum β-lactamase in a neonatal intensive care unit. Korean J Healthc Assoc Infect Control Prev.

[23] Lenglet A, Faniyan O, Hopman J. A nosocomial outbreak of clinical sepsis in a neonatal care unit (ncu) in Port-Au-Prince Haiti, july 2014 - september 2015. PLoS Curr. 2018;10. 10.1371/currents.outbreaks.58723332ec0de952adefd9a9b6905932. 10.1371/currents.outbreaks.58723332ec0de952adefd9a9b6905932 PMC5866103 29637010

[24] Cha MK, Kang CI, Kim SH, Chung DR, Peck KR, Lee NY (2018). High prevalence of ctx-m-15-type extended-spectrum beta-lactamase among ampc beta-lactamase-producing klebsiella pneumoniae isolates causing bacteremia in Korea. Microb Drug Resist.

[25] Mavroidi A, Liakopoulos A, Gounaris A, Goudesidou M, Gaitana K, Miriagou V (2014). Successful control of a neonatal outbreak caused mainly by st20 multidrug-resistant shv-5-producing klebsiella pneumoniae, Greece. BMC Pediatr.

[26] Dumpis U, Iversen A, Balode A, Saule M, Miklasevics E, Giske CG (2010). Outbreak of ctx-m-15-producing klebsiella pneumoniae of sequence type 199 in a latvian teaching hospital. APMIS.

[27] Mshana SE, Hain T, Domann E, Lyamuya EF, Chakraborty T, Imirzalioglu C (2013). Predominance of klebsiella pneumoniae st14 carrying ctx-m-15 causing neonatal sepsis in Tanzania. BMC Infect Dis.

[28] Marcade G, Brisse S, Bialek S, Marcon E, Leflon-Guibout V, Passet V (2013). The emergence of multidrug-resistant klebsiella pneumoniae of international clones st13, st16, st35, st48 and st101 in a teaching hospital in the Paris region. Epidemiol Infect.

[29] Peltier F, Choquet M, Decroix V, Adjide CC, Castelain S, Guiheneuf R (2019). Characterization of a multidrug-resistant klebsiella pneumoniae st607-k25 clone responsible for a nosocomial outbreak in a neonatal intensive care unit. J Med Microbiol.

[30] Ahn S, Sung JY, Kim H, Kim MS, Hwang Y, Jong S (2016). Molecular epidemiology and characterization of carbapenemase-producing enterobacteriaceae isolated at a university hospital in Korea during 4-year period. Ann Clin Microbiol.

[31] Jazmati N, Jazmati T, Hamprecht A (2017). Importance of pre-enrichment for detection of third-generation cephalosporin-resistant enterobacteriaceae (3gcreb) from rectal swabs. Eur J Clin Microbiol Infect Dis.

